# Tuberculosis patients’ pre-hospital delay and non-compliance with a longstanding DOT programme: a mixed methods study in urban Zambia

**DOI:** 10.1186/s12889-016-3771-9

**Published:** 2016-10-28

**Authors:** Anne Lia Cremers, René Gerrets, Nathan Kapata, Austin Kabika, Emma Birnie, Kerstin Klipstein-Grobusch, Martin P. Grobusch

**Affiliations:** 1 Center of Tropical Medicine and Travel Medicine, Department of Infectious Diseases, Division of Internal Medicine, Academic Medical Center, University of Amsterdam, Amsterdam, The Netherlands; 2 Faculty of Social and Behavioural Science, Department of Sociology and Anthropology, University of Amsterdam, Amsterdam, The Netherlands; 3 National TB and Leprosy Control programme, Lusaka, Zambia; 4 University of Zambia - University College London (UNZA-UCL) programme, Lusaka, Zambia; 5 Department of Psychology, School of Humanities and Social Sciences, The University of Zambia, Lusaka, Zambia; 6 Julius Global Health, Julius Center for Health Sciences and Primary Care, University Medical Center Utrecht, Utrecht, The Netherlands; 7 Division of Epidemiology and Biostatistics, School of Public Health, Faculty of Health Sciences, University of the Witwatersrand, Johannesburg, South Africa

**Keywords:** Tuberculosis, Public health, Pre-hospital delay, Treatment compliance, Zambia

## Abstract

**Background:**

Tuberculosis (TB) remains a major health problem in Zambia, despite considerable efforts to control and prevent it. With this study, we aim to understand how perceptions and cultural, social, economic, and organisational factors influence TB patients’ pre-hospital delay and non-compliance with care provided by the National Tuberculosis Programme (NTP).

**Methods:**

A mixed methods study was conducted with 300 TB patients recruited at Kanyama clinic for structured interviews. Thirty were followed-up for multiple in-depth interviews. Six focus group discussions were organised and participant observation was conducted. Ten biomedical care providers, 10 traditional healers, and 10 faith healers were interviewed. Factors associated with non-compliance (disruption of treatment > one week) were assessed by applying logistic regression analyses; qualitative analysis was used to additionally assess factors influencing pre-hospital delay and for triangulation of study findings.

**Results:**

TB treatment non-compliance was low (10 %), no association of outcome with cultural or socio-economic factors was found. Only patients’ time constraints and long distance to the clinic indicated a possible association with a higher risk of non-compliance (OR 0.52; 95 % CI 0.25, 1.10, *p* = 0.086). Qualitative data showed that most TB patients combined understandings of biomedical and traditional TB knowledge, used herbal, traditional and/or faith healing, suffered from stigmatizing attitudes, experienced poverty and food shortages, and faced several organisational obstacles while being on treatment. This led in some cases to pre-hospital delay or treatment non-compliance.

**Conclusions:**

Mixed methods analysis demonstrated the importance of in-depth information ascertained by qualitative approaches to understand how cultural, socio-economic and organisational factors are influencing patients’ pre-hospital delay and treatment compliance. To strengthen the Zambian NTP, combating stigma is of utmost priority coupled with programmes addressing poverty. Organisational barriers and co-operation between (private) clinics and traditional/faith healers should be considered.

**Electronic supplementary material:**

The online version of this article (doi:10.1186/s12889-016-3771-9) contains supplementary material, which is available to authorized users.

## Background

Tuberculosis (TB) is one of the major global health problems causing morbidity and mortality worldwide [1], with the highest death rates in sub-Saharan Africa [2]. In Zambia, the estimated incidence of tuberculosis is 427/100.000; 61 % of TB patients are co-infected with the human immunodeficiency virus (HIV), and multi-drug resistant TB (MDR-TB) is found in 0.3 % of new cases and in 8.1 % of retreatment cases [1].

In 1993, the World Health Organization (WHO) responded to this severe public health threat by promoting the Directly Observed Therapy (DOT) policy to ensure patients’ compliance with their treatment [3]. However, DOT is often criticized for its paternalistic nature, and its implementation by National TB Programmes (NTPs) is often complicated by socio-economic factors [4]. Current public health approaches to control TB infection often take into account the co-prevalence of MDR-TB and HIV infections, pill burden, long treatment intervals, overburdened TB control programmes, and generally also consider cultural and socio-economic factors influencing health care seeking behaviour of TB patients [4–9]. Often, vulnerable populations are afflicted as TB is closely related to issues of stigma, (economic) inequality, and poverty [5]. Stigma often leads to the discrimination or social exclusion of TB patients, negatively influencing their health care seeking behaviour [10]. Poverty complicates access to health care as people are unable to stop working or to pay for public transport; and hunger aggravates side effects of TB treatment. Moreover, children with TB are difficult to diagnose and are often under-represented in NTPs [11–14].

The worrisome situation in Zambia has triggered the development of various TB strategies and public health efforts [15, 16]. Despite a longstanding DOT and sensitisation programmes, Zambia still struggles with optimizing its NTP to avoid pre-hospital delay and treatment non-compliance of TB patients. Most studies reporting on the TB policy and care provision in Zambia use quantitative methods [17–22], focus on HIV-TB co-infection [19, 20, 23, 24], are located in rural Zambia [19–21, 23–25], or do not take children with TB into account [17–21, 24]. To the best of our knowledge no study has been published focussing specifically on pre-hospital delay and treatment non-compliance of TB patients (including children) in urban Zambia using a combination of qualitative and quantitative methods.

The first analysis of our TB patients’ Adherence and Compliance (TBAC) study focused on TB-related stigma in Lusaka, Zambia [10]; the current analysis aims to investigate the influence of perceptions and cultural, social, economic, and organisational factors on TB patients’ pre-hospital delay and compliance with care provided by the Zambian NTP.

## Methods

### Setting

The study took place at Kanyama clinic in the urban township Kanyama in Lusaka, Zambia, from September 2013 to January 2014. Details on Kanyama (clinic) and the tuberculosis programme have been reported elsewhere in detail [10].

### Mixed methods

We used a mixed methods approach and a sequential explanatory model in which quantitative and qualitative research techniques were given equal priority; highlighting different aspects of the study. The mixed methods approach allowed for triangulation of study findings.

### Study population and collection of data

During four months, we conducted researcher-administered structured interviews (each approximately 30 min) with 300 TB patients attending Kanyama clinic for TB treatment. The sample size allowed us to estimate nine parameters of treatment compliance in a multivariate logistic regression, based on a prevalence of patients lost-to-follow-up (LTFU) of 30 % in a Zambian study [22] and the sample size recommendations for a logistic regression analysis to investigate predictors with a 95 % confidence interval [26]. Patients who paused their treatment for one or more weeks were considered non-compliant and those who had abandoned treatment for two weeks or more were additionally considered LTFU.

Subsequently, of those 300 patients, we randomly followed-up on 30 patients for one to three in-depth semi-structured interviews (one to two hours each) at their homes. To enhance our understanding of the TB programme and the social and cultural context, we conducted in-depth interviews with ten TB health workers, ten faith healers, and ten traditional healers located in Kanyama using a convenience sample.

Additionally, we organised six focus group discussions (FGDs). Through convenience sampling, we recruited 10 treatment supporters for the first FGD and for the other five FGDs eight patients and two treatment supporters each. We conducted participant observation at the clinic, the TB department, patients’ homes/neighbourhoods, clinics/homes of traditional healers, and during faith healing sessions in various Christian churches in Kanyama district.

We designed a structured questionnaire to discuss demographics, biomedical knowledge, treatment history, treatment compliance, and TB-related difficulties. The in-depth interviews elaborated on the questionnaire and discussed sensitive or complex topics, such as stigma and pre-hospital delay. Patients who had waited over four weeks since the onset of symptoms prior to their initial hospital visit were considered to have a pre-hospital delay [27]. The FGDs covered the topics (1) challenges in the work of TB lay health workers; (2) childhood TB-related difficulties; (3) TB-related struggles; (4) stigma; (5) health care seeking; and (6) biomedical knowledge about TB. We used techniques to evoke discussions and make respondents comfortable to speak their minds; such as word association games, ranking of themes, and poster presentations. The semi-structured in-depth interviews with TB health workers, traditional and faith healers elaborated on TB care and their work-related challenges.

### Statistical outcomes, variables and analysis

We compared demographics and TB treatment-related parameters of the in-depth study sample (*N* = 30) with the larger study group of TB patients (*N* = 270). We used the Fisher’s exact test for categorical data and the Student t-tests for normally distributed continuous data. To identify factors that could be associated with TB patients’ treatment non-compliance, we conducted logistic regression analyses. We conducted analysis with IBM SPSS statistics version 21.0 (IBM Corp, Armonk, NY).

### Qualitative outcomes, thematic, and content analysis

Qualitative data was analysed to explain, contextualize and interpret quantitative findings. For the in-depth interviews and FGDs, we conducted thematic and content analysis assisted by Qualitative Data Analysis and Research Software (ATLAS.ti, 7th edition; Scientific Software Development GmbH, Berlin, Germany). Transcripts were screened multiple times, coded into meaning units and categorized into broad themes [28]. Additionally, we analysed context, meaning, and structures of identified codes and themes [29]. Some quotes of respondents were used to illustrate the most important themes [10].

## Results

### Study group

Mean age of the study population (*N* = 300) was 33 years (range 1–70 years), including 25 children and teenagers under the age of 20. In total, 193 patients were male (64 %); 179 (59.7 %) were in a relationship; 126 (42 %) had attended seven years of state-funded primary school; and 205 (68 %) were (self-)employed. Patients were on average 11 weeks on treatment [range 1–52]; 86 (29 %) relapsed, i.e. were diagnosed again with active TB after TB treatment completion (some repeatedly); 274 (94 %) had done Voluntary Counselling and Testing (VCT) for HIV; and 147 (54 %) had a positive HIV status of which 101 (69 %) were on antiretroviral therapy (ART). For the qualitative sample, six children/adolescents and 24 adults were in-depth interviewed with a mean age of 31 years [range 2–54]; of which 17 (57 %) were male. Demographics and tuberculosis-related parameters of the in-depth sample were similar between the in-depth and quantitative sample (Table 1).

**Table 1:** Patient characteristics (300 TB patients), comparison quantitative sample *N* = 270 with in-depth sample *N* = 30, Lusaka, Zambia

Variable	Overall study group *N* = 300	Quantitative sample *N* = 270	In-depth sample *N* = 30	*P*-value^a^	OR (95 % CI)^b^
	*N* (%)^c^	*N* (%)	*N* (%)		
Age (years)					
Mean [SD]	33.3 [11.3]	33.6 [11.1]	31.0 [13.3]	0.240	(−6.85, 1.72)
Sex					
Male	193 (64.3)	176 (65.2)	17 (56.7)	0.422	1.43 (0.67, 3.08)
Female	107 (35.7)	94 (34.8)	13 (43.3)		
Marital status					
Married/relation	179 (59.7)	165 (61.1)	14 (46.7)	0.169	1.80 (0.84, 3.83)
Single	121 (40.3)	105 (38.9)	16 (53.3)		
Education					
Low (none-7 years)	148 (49.3)	136 (50.4)	12 (40.0)	0.337	1.52 (0.71, 3.28)
High (7 yrs-higher)	152 (50.7)	134 (49.5)	18 (60.0)		
Profession					
Employed	205 (68.3)	187 (69.3)	18 (60.0)	0.307	1.50 (0.69, 3.26)
Unemployed	95 (31.7)	83 (30.7)	12 (40.0)		
Treatment duration (wks)					
Mean [SD]	10.8 [8.3]	10.9 [8.3]	9.5 [9.0]	0.368	(−4.60, 1.71)
TB Relapse					
Yes	86 (29.0)	77 (28.8)	9 (30.0)	1.000	1.06 (0.46, 2.41)
No	211 (71.0)	190 (71.2)	21 (70.0)		
Unknown	3 (1.0)	3 (1.1)	0 (0.0)		
VCT					
VCT	274 (94.2)	247 (94.3)	27 (93.1)	0.681	1.22 (0.27, 5.62)
No VCT	17 (5.8)	15 (5.7)	2 (6.9)		
Unknown	9 (3.0)	8 (3.0)	1 (3.3)		
HIV					
HIV positive	147 (53.8)	130 (52.8)	17 (63.0)	0.417	0.66 (0.29, 1.50)
HIV negative	126 (46.2)	116 (47.2)	10 (37.0)		
Unknown	27 (9.0)	24 (8.9)	3 (10.0)		
HIV patients on ART					
ART	101 (68.7)	86 (66.2)	15 (88.2)	0.094	0.26 (0.06, 1.19)
No ART	46 (31.3)	44 (33.8)	2 (11.8)		
Unknown/not applicable	153 (51.0)	140 (51.9)	13 (43.3)		

^a^Fisher’s Exact Test for categorical variables, *t*-test for continuous variables comparing quantitative and in-depth sample

^b^OR (95 % CI) Odds Ratio and 95 % Confidence Interval

^c^Valid percent

### Statistics and TB treatment non-compliance

**Fig. 1:**
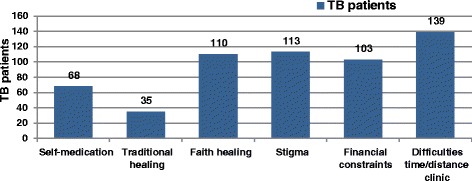
TB-related struggles and alternatives for biomedical TB care of 300 TB patients, Lusaka, Zambia. Numbers do not add up to 300, because the questions allowed for multiple responses

In total, 31 patients (10 %) had previously been non-compliant, ranging from pausing treatment for a week to completely abandoning treatment (LTFU). Patients mentioned one or more of the following reasons: feeling better (4 %), side effects (2 %), being physically and financially unable to come to the clinic (2 %), inability to buy food (1 %), drinking beer (1 %), or having switched to faith healing in a Christian church or herbal healing (1 %). Overall, patients mentioned the use of alternative health care, such as faith healing (36.6 %), self-medication (22.7 %), and traditional healing (11.7 %), and struggles with TB care, such as stigma (37.7 %), financial constraints and hunger (34.3 %), and/or difficulties with time constraints and/or long distances to be covered to reach the clinic (46.3 %) (Fig. 1).

There was no evidence of an association between non-compliance and sex, age, education, profession, HIV, seeking alternative health care, stigma, or financial constraints (Table 2). Patients’ difficulties with time/distance to be at/reach the clinic indicated a possible association with a higher risk of non-compliance (OR 0.52; 95 % CI 0.25, 1.10, *p* = 0.086). The majority (76 %) came on foot to the clinic (time ranged from five minutes up to two hours depending on distance and ability to walk). Many patients declared drug collection constraints with time and distance to the clinic due to work, travel, domestic responsibilities, financial hurdles, or physical inability to walk.

**Table 2:** Univariate logistic regression analyses of factors associated with treatment non-compliance^a^ of 300 TB patients, Lusaka, Zambia

Variable	Compliant *N* = 269	Non-compliant *N* = 31	*P*-value	OR (95 % CI) ^b^
	*N* (%) ^c^	*N* (%) ^c^		
Sex (male)	169 (63.3)	24 (72.7)	0.230	0.60 (0.26, 1.39)
Age (>31 years^d^)	151 (56.1)	16 (51.6)	0.632	1.20 (0.57, 2.52)
Level of education (none - primary school)	133 (49.8)	15 (45.5)	0.624	0.83 (0.39, 1.75)
Profession (employed)	179 (67.0)	26 (78.8)	0.255	0.60 (0.25, 1.45)
HIV (HIV +)	129 (53.3)	18 (58.1)	0.586	1.24 (0.57, 2.71)
Seeking alternative health care (yes)	138 (51.7)	16 (48.5)	0.729	1.14 (0.54, 2.40)
Stigma (yes)	101 (82.1)	12 (80.0)	0.841	0.87 (0.23, 3.35)
Financial constraints (yes)	89 (33.3)	14 (42.4)	0.348	0.70 (0.33, 1.48)
Difficulties with time/distance to be at/reach the clinic (yes)	104 (39.0)	17 (51.5)	0.086	0.52 (0.25, 1.10)

^a^Patients who had previously paused their treatment for one or more weeks or had abandoned treatment were considered non-compliant

^b^OR (95 % CI): Odds Ratio and 95 % Confidence Interval

^c^Valid percent

^d^In both age groups same percentage of non-compliant patients

### TB perceptions

In total, 175 TB patients (58 %) explained they had no previous biomedical knowledge before coming to the clinic; and 198 (66 %) did not associate their symptoms with TB beforehand. Forty-five patients got biomedical TB information at school (15 %), 180 from friends or family (60 %), and/or 201 at the clinic (67 %). In total, 99 patients (33 %) said they had not received any information at the clinic. Some explained that they had been too tired for the sensitisation talks of the TB health workers or that the information was too difficult to comprehend.

A majority of 293 patients (98 %) was able to mention one or more of the four main TB symptoms highlighted in the clinic’s sensitisation brochure: coughing, night sweats, loss of appetite/weight, and chest pain. When asked about the consequences of interrupted or incorrect drug intake, 51 patients (17 %) had no idea; 79 (26 %) mentioned resistance to TB drugs, and 170 (57 %) referred to death, injections, restart of treatment, and/or falling ill, but were generally unsure about the reason why this would happen. The biomedical aetiology of TB was known by 286 patients (95 %) who mentioned its airborne nature and/or coughing. Some patients used biomedical terms, but after probing they did not fully comprehend the meaning. Moreover, 270 patients (90 %) combined biomedical explanations (cough/airborne) with one or more alternative aetiological principles. A considerable group of patients stated that TB could be contracted by sharing a cup or plate with a TB patient (52 %) or that TB could (also) be caused by evil spirits or witchcraft (28 %). Some respondents claimed that TB was a genetic disease (*family TB*), a disease caused by God or fallen angels, or by immoral behaviour, such as drinking beer, smoking, promiscuity and prostitution (Table 3).

**Table 3:** Biomedical TB knowledge reported by 300 TB patients during researcher-administered structured interviews at Kanyama clinic, Lusaka, Zambia

		*N* (%) ^a^
Symptoms		
Respondents who knew TB symptoms: coughing, night sweats, loss of appetite, chest pain	no symptoms of TB	7 (2.3)
	two or three symptoms of TB	207 (69.0)
	four symptoms of TB	86 (28.7)
Treatment compliance and multi-drug resistant TB		
Respondents who	did not know the importance of treatment compliance	51 (17.0)
	knew the importance of treatment compliance	170 (56.7)
	knew the importance of treatment compliance and understood the meaning of DR-TB	79 (26.3)
TB aetiologies		
Respondents who mentioned	airborne	257 (85.7)
	cough	233 (77.7)
	sharing cups, utensils	155 (51.7)
	evil spirits/witchcraft	85 (28.3)
	traditional myths	58 (19.3)
	smoking	50 (16.7)
	drinking beer	39 (13.0)
	promiscuous behaviour	38 (12.7)
	prostitution	32 (10.7)
	god	28 (9.3)
	genetic disease (Family TB)	11 (3.7)

^a^Numbers do not add up to 300, because the questions allowed for multiple responses

During in-depth interviews and FGDs, various respondents explained that coughs in general, and TB more specifically, could be explained with the local term *traditional myths*. According to such myths, a cough was caused by eating food that had been salted by a menstruating woman or a woman who had just aborted; or when having sexual intercourse with a menstruating woman or a woman who had just aborted. In the latter case, the spirit of an aborted embryo (*Kapopo)* caused the cough. Some respondents also mentioned that babies with a cough are suspected to have been *tyoled –* a locally used term indicating that their father had been unfaithful and touched the baby in the first week after birth.

When asking about the links between *traditional coughs* and TB, responses varied. Some patients denied any linkage:

*I don’t believe you can get TB according to this myth about abortion or menses, but you can get Chantanda wanga* [a cough] *if you do that. The kapopo* [aborted embryo] *comes to you, just like that, but it is not TB* (in-depth interview 52-year old male TB patient).

Most traditional healers and several TB patients made a distinction between heavy, deep coughs that can be explained by a traditional myth, versus normal coughs caused by TB. Health workers said that some patients didn’t believe their cough was related to TB. This disbelief was even more prevalent in case of children, because many people explained that children were unable to get TB and that their cough had to be caused by witchcraft or by being *tyoled*. Some patients attributed both myths and biomedical aetiologies to TB infection combining the information of clinical staff with the local knowledge about traditional myths. Some patients explained that any cough, and therefore also the traditional coughs could indicate TB infection. Health workers shared this message during community sensitisation programmes. Various patients were uncertain about the relation between traditional coughs and TB.

### Cultural factors

In total, 154 patients (51.3 %) reported concurrent use of biomedical health care provision, such as traditional, faith, and self-healing (Tables 2 and 4). During FGDs, respondents explained that many community members did not start with a clinic visit, but rather used locally available herbal treatments in Kanyama to treat their cough. Generally, the second step was going to a market or *Kantemba*, a cheap unlicensed pharmacy.

**Table 4:** Alternative healing methods for tuberculosis in Urban Zambia assessed both during researcher-administered structured interviews, in-depth interviews with TB patients, traditional and faith healers, and FGDs at Kanyama clinic

Healing methods	Medication or practices
Self-medication - herbal	Lemons, ginger, garlic, beetroot, leaves of: the banana plant, the Moringa, Nim, Blue Gum eucalyptus, guava, or mango tree, aloe Vera
Self-medication - Katemba / markets	Panadol, cough syrup, Chinese medicine*, Back-to-Eden-herbs*
Faith healing	Prayer, deliverance (exorcism of evil spirit), fasting and praying on a sacred mountain, holy water and/or anointed oil, *Back-to-Eden-herbs,* faith
Traditional healing - herbal	Traditional herbs, such as *Nkonka, Muleza* (also named *Kankalamba* or *Munsokansoka*)*, Mutato* (herbal energy booster)
Traditional healing - spiritual	Witchcraft (*mfuiti*), consulting the spirits/ancestors, sacrificing small animals

During FGDs, participants explained that the majority of Zambians generally used traditional healing for treating illness, yet seldom for TB. This idea was slightly nuanced during in-depth interviews where often a difference was made between traditional healers in the city and in the village, making some of them travel to rural areas for TB care:

*For TB I don’t like African herbs or pastors. Many people have died. […] Traditional healers steal your money. Only some will know everything, if you are bewitched and by whom. But nowadays many doctors just make it up. The real traditional healers from the village can heal TB, but they’re few* (in-depth interview, 28-year old male TB patient).

Some respondents had initially started with faith healing. Respondents favouring faith healing often said that TB resulting from evil spirits could only be healed by faith healing and not in the clinic.

*At church, people say spirits bring disease. A demon for TB, a demon for HIV. If you pray, you get healed. I also believe in that. But for my daughter it is not a demon, because deliverance* [exorcism of disease-bringing spirit] *didn’t help. Then we went to the clinic* (in-depth interview 33-year old mother of nine year-old TB patient).

During in-depth interviews and one FGD, patients mentioned reasons for not first attending Kanyama clinic when falling ill, such as the idea they had a general cough; that TB was caused by non-biomedical causes; advice of their pastor or family member; or advantages from attending alternative care providers such as absence of long queues, quick patient service, guaranteed anonymity, and easier accessibility. Some patients and biomedical staff explained that traditional healers and faith healers could cause substantial pre-hospital delays.

### Social factors

During structured interviews, 113 TB patients (35 %) mentioned they suffered from loss of self-esteem, shame, insulting remarks, ridicule, discrimination, divorce, dismissal at work, and/or social isolation. Yet during in-depths interviews at home, more than half of the patients (60 %) elaborated on the consequences of stigmatizing neighbours and friends.

*I had TB before, and my neighbours said to their children: don’t go in that house! They never come near me, only greet me from far away. They are afraid of getting TB. They will say: that is not a normal cough. That is TB. He has Kanayaka* (in-depth interview 24-year female TB patient).

During the FGD on TB-related stigma, both patients and TB health workers explained to me that the local derogatory term for people with HIV, *kanayaka* – literally, “*the red light that does not switch off*” – is often used to label TB patients. The label *kanayaka* signalled to others that TB patients were infectious and therefore dangerous, and that their lives would soon come to an end. Because of the label many TB patients explained that they additionally dealt with HIV-associated negative stereotyping, being accused of immoral behaviour, promiscuity, alcoholism, chain-smoking, and prostitution.

The FGDs also provided insight in the relation between stigmatization and a denial of a positive TB diagnosis, non-disclosure, and/or difficulties with initial hospital visit and treatment compliance. Fearful of a positive diagnosis and associated stigmatizing reactions, some people rejected testing:

*One of my cousins* [a household member] *died of TB, because she didn’t want to go to the clinic and didn’t want treatment. I have tested myself and my grandson. The rest of the household doesn’t want to do a test for TB or HIV, because they are afraid for the test results. They say they can’t have TB* (interview 65-year old grandmother of 1-year old child on preventive TB treatment).

Respondents explained that women were sometimes forcibly expelled to the village to hide their TB diagnosis and treatment from neighbours. (No answer was given to why this did not happen for men.) During one in-depth interview, a TB patient described that she was send away and that in rural areas TB treatment was not easily accessible or available. This contributed to her treatment disruption and aggravation of TB. She had almost died and came back to the city to attend Kanyama clinic and retake TB treatment.

### Economic factors

Quantitative data showed that 103 patients (34 %) faced serious food shortages.

*In Zambia the problem is mostly food. You eat once a day, it is not even good. Especially with TB drugs, it is hard, every day we have to take drugs. It makes you hungry, dizzy, shaky, but I have no food, no money* (interview 37-year old male TB patient).

The in-depth interviews provided insight into the intersection of poverty and treatment compliance. Patients explained that the “strong medication” made them feel hungry and weak. Hunger complicated coping with side effects and treatment compliance. Some patients filled their stomach with cheap maize opaque beer (*Chibuku*), generally not realising that alcohol can render TB medications less effective. Patients explained that alcohol was also a way to free their minds of (financial) problems.

During in-depth interviews, health care workers and various patients indicated delays in search for TB care at the clinic, because poverty forced many patients to continue working and the majority had no health insurance. 136 patients (45.3 %) were in a severe health condition on first arrival at the clinic. Few patients were financially supported by their family-members, employers, or landlords.

### Organisational factors

Participant observation and informal conversations with both the TB health workers and the patients provided insight into the efforts of the understaffed TB team regarding provision of patient service, medication distribution, and record-keeping at the TB department of Kanyama clinic. The voluntary treatment supporters seemed an indispensable asset enhancing contact between TB patients and the clinic and sensitizing the community about TB infection. Moreover, the nurses explained that in the past the clinic had contacted several faith healers and three private clinics in Kanyama district in order to enhance hospital referral of TB patients, and to supply the latter with correct TB drugs. The Kanyama clinic worked with a strict TB drug collection schedule based on the stage of treatment patients were involved in. Consequently, family members in different treatment stages were required to individually collect medication on different days.

Participant observation showed that patients simultaneously diagnosed with TB and HIV received additional care at the clinic (different medication, support groups); however, three HIV patients who started TB treatment at a later stage went unnoticed by health workers, leading to a mismatch of TB and HIV drugs. Other organisational problems regarding administration of TB patients occurred when patients moved to the rural area and were transferred to a local clinic. These patients were invariably LTFU.

## Discussion

In this study, we examined perceptions and cultural, social, economic, and organisational factors influencing TB patients’ pre-hospital delay and compliance to care provided by the NTP using a mixed methods research approach. Quantitative data analysis showed no association between treatment compliance and demographic characteristics, patients’ TB-related struggles, or alternative health care seeking. In contrast, qualitative data identified how TB perceptions, stigma, poverty, and organisational obstacles influenced TB patients’ pre-hospital delay and treatment non-compliance undermining an effective TB control programme.

### TB perceptions

Quantitative findings illustrated that most TB patients used both biomedical and traditional understandings of TB knowledge. Qualitative findings provided insight that many patients were unsure how to combine the variety of TB perceptions available. Previous studies have shown the importance of perceptions on health care seeking behaviour [28–32] and have stressed the importance of cultural-sensitive sensitisation programmes [7, 8, 33]. We propose interactive awareness programmes that acknowledge and appropriately address the variety of local perceptions to enhance early case-finding and reduce hospital delay. Furthermore, patients’ usage of traditional healers, faith healers, and private clinics calls for a collaborative strategy between clinics and these alternative healers, as promoted by the WHO [34] and proven effective according to various studies in sub-Saharan Africa [35–38].

### Stigma

Quantitative analysis showed a high prevalence of stigmatising attitudes and actions by patients’ community and family members, as we have previously reported in detail [10]. Stigmatizing perceptions were amongst others represented by the locally-used derogatory term *Kanayaka,* to label both TB and HIV patients. This linkage seemed to aggravate TB patients’ experience of stigma, targeting them additionally with HIV-related accusations. This is in line with previous reports from Zambia [3, 25]. Children were as vulnerable to stigma as adults calling for more research focused on this age group [10]. A high number of the stigmatised patients in this study consisted of women, whose vulnerable position in society was exacerbated by the use of local traditional myths blaming women for the spread of TB and HIV [3, 10]. TB programmes targeting stigma-related perceptions and attitudes need further improvement [10], specifically regarding patient support and family sensitisation, which could be achieved through organisation of support groups for TB patients irrespective of HIV status. Moreover, the collaborative TB/HIV programme should address the extra dimension of TB-related stigma linked to HIV with renewed TB/HIV sensitisation programmes and pay particular attention to the vulnerable position of children and women herein.

### Poverty

Patients’ low socio-economic status was related to often cited struggles of regular food shortages, resonating with World Bank statistics [39] that 61 % of the Zambian population lives below the poverty line. A case study from Lusaka described poverty levels to be specifically high in slum areas such as Kanyama [40]. In-depth interviews with health care staff and patients clarified that almost none of the respondents had a private health insurance. Consequently, patients often postponed an initial clinic visit or (temporarily) discontinued treatment to avoid loss of income. When falling seriously ill, many patients faced (financial or physical) difficulties attending the clinic, taking treatment, and/or deal with adverse medication effects. TB often disrupted livelihoods as cash income declined, especially when breadwinners fell ill.

Participant observation showed that TB patients lived in conditions with a high risk of TB infection due to overcrowded housing and poor ventilation. The majority of people in the community were too poor to reduce these risks, and given high local crime rates, many patients preferred closed doors and windows. Because the TB programme is mainly focused on physical health and not on improving living conditions, cured patients can acquire TB again and again. Poverty is well known to fuel tuberculosis and remains a major challenge to TB control programmes worldwide, undermining the effectiveness of (free) TB drugs [5, 41–44]. Several Zambian studies have described low socio-economic status to be an obstacle to TB care referring to the necessity of food aid and payment of transport costs for both TB and HIV patients [21, 23, 45, 46]. About a decade ago, two Zambian studies described successful Home Based Care (HBC) organisations [47, 48] providing food aid, but due to financial constraints these organisations ceased their programmes. Based on our qualitative findings and the literature, we advocate to not only diminish symptoms of poverty with renewed HBC programmes, but to also make an end to what may be considered structural violence embedded within longstanding social and economic structures of inequality [49]. The position of TB patients and associated TB prevalence and incidence, can only be improved if poverty is substantially alleviated.

Alcohol consumption was often mentioned by respondents in relation to poverty. Sensitisation programmes about alcohol’s destructive effects on TB treatment fall short, ignoring the socio-economic context in which TB patients in our study sometimes depended on alcohol to deal with hunger, (financial) problems, and to numb themselves. Data from South Africa showed that poverty alleviation reduced substance abuse among South African TB patients and improved TB care [50].

### Organisational obstacles

Difficulties with TB patients’ long time/distance to the clinic’ indicated a trend with ‘treatment non-compliance’, which could potentially relate to patients’ work-related commitments making it difficult to (timely) collect medication. The clinic should take time and distance constraints into consideration permitting patients to collect medication for a longer period of time and/or for the whole household, eliminating long queues and enhancing a direct flow to the TB department possibly preventing unnecessary deaths and in-hospital transmission.

Furthermore, full integration of TB and HIV services fell short in certain instances and is of utmost importance; as recommended by the national TB control programme; and as implemented in other sub-Saharan African countries [51–53]. Moreover, inadequate communication between Kanyama clinic and rural clinics hindered record keeping of transferred patients and an accurate administration and are in need of improvement.

### Limitations and strengths

A limitation of this study was that pre-hospital delay was only qualitatively and not quantitatively assessed, because related structured interview questions triggered vague or contradictory responses due to lack of time, and inability of interviewees to remember in detail their pre-hospital illness experience. In-depth interviews did generate insights regarding pre-hospital delay, suggesting future qualitative research on the topic. Furthermore, it was impossible to add TB knowledge as a risk factor for treatment compliance in the univariate analysis, because of the time gap between previous non-compliant behaviour and current knowledge. Prospective studies are needed assessing the impact of baseline TB knowledge on treatment non-compliance.

The strengths of this study was the mixed method design and sequential explanatory model. Triangulation of research findings enabled iterative analysis. The combination of methods was specifically valuable when quantitative methods fell short and qualitative methods could fill these information gaps.

## Conclusions

Mixed methods analysis clearly demonstrated the importance of qualitative approaches to understand how cultural, social, economic and organisational factors are influencing pre-hospital delay and treatment non-compliance with regards to the Zambian NTP. Patients’ concurrent use of local TB understandings and health care calls for cultural sensitive TB education and co-operation between (private) clinics and traditional/faith healers. To strengthen the Kanyama clinic’s existing programmes, combating stigma is of utmost priority coupled with programmes addressing poverty. Organisational barriers regarding drug collection schedules, patient transfers, and integrated HIV-TB programme should be addressed.

## Additional file

Additional file 1: Dataset quantitative part TBAC study. (XLS 230 kb)

## Abbreviations

ART: Antiretroviral therapy
DOT: Directly observed therapy
FGD: Focus group discussion
HBC: Home based care
HIV: Human immunodeficiency virus
LTFU: Lost-to-follow-up
MDR-TB: Multi-drug resistant tuberculosis
NTP: National Tuberculosis Programme
TB: Tuberculosis
TBAC: TB patients’ adherence and compliance
VCT: Voluntary counselling and testing for HIV
WHO: World Health Organization
